# A study of Galactin-3 on fine needle aspiration as a diagnostic marker differentiating benign from malignant thyroid neoplasm

**DOI:** 10.12669/pjms.333.12251

**Published:** 2017

**Authors:** Alliya Muzafar, Mulazim Hussain Bukhari, Ihtesham uddin Qureshi

**Affiliations:** 1 Dr. Alliya Muzafar, MBBS, M.Phil. Senior Demonstrator Pathology, King Edward Medical University, Lahore, Pakistan; 2 Prof. Dr. Mulazim Hussain Bukhari, MBBS, FCPS, PhD. Head of Department Pathology, University Medical College, University of Lahore, Lahore, Pakistan; 3 Prof. Dr. Ihtesham uddin Qureshi, MBBS, M.Phil. Professor of Pathology, Postgraduate Medical Institute, Lahore, Pakistan

**Keywords:** Galactin-3, Follicular Adenoma, Follicular Carcinoma, Papillary Carcinoma

## Abstract

**Background & Objective:**

Thyroid nodules are very common in our setup and their diagnosis on fine needle aspiration is not easy and is a taxing affair. It is a challenge to differentiate between follicular adenoma and follicular carcinoma without histology. Our objective was to investigate the role of Galectin-3 in fine needle aspirates of thyroid nodules as a prospective diagnostic marker and consequently its ability to differentiate benign from malignant neoplasms.

**Methods:**

The research was conducted at the department of Pathology, King Edward Medical University, in association with other teaching institutions of Lahore from June 2012 to July 2014.. Sixty cases of solitary thyroid nodules were included in the study. Haematoxylin and eosin staining of the fixed smears and Galectin-3 immunohistochemical staining of the sections prepared from the cell block was performed.

**Results:**

There were 60 patients in our study with a mean age of 33.35 years. The Bethesda system for reporting thyroid cytopathology was used to classify the smears and only categories IV, V and VI were included. On histological examination of the resected nodules there were 38.3% (23/60) cases of follicular adenoma, 46.6% (28/60) were of papillary carcinoma and follicular carcinoma made up to 15% (9/60) of all cases. Galectin-3 was negative in 100% (23/23) cases of follicular adenomas. Out of 37 malignant cases 65% lesions showed positivity, while 35% showed negativity for this immunomarker. Considering the malignant lesions, 75% cases of papillary carcinomas showed a positive reaction while only 33% of follicular carcinomas were positive for the immunomarker. This showed that the positive expression was more common in papillary as compared to follicular carcinomas.

**Conclusion:**

Galectin-3immunomarker is considerably expressed in malignant tumors, but it is not expressed in benign follicular lesions.

## INTRODUCTION

Fine needle aspiration and cytology (FNAC) has become a commonly used technique in the clinical management of TN.^1^ It is considered as a useful diagnostic aid as well as a screening test in this condition with an overall accuracy of around 95% in detection of thyroid cancers.^2^ In the process of evaluation of nodular lesions of thyroid, FNAC remains the gold standard and is definitely the procedure of choice. However, FNAC has its shortcomings and diagnostic drawbacks like any other test. Major issues are the gray-zoned cases i.e. cases suspicious for malignancy.^3^

It has been reported in different studies that CD44 and Galectin-3 can serve as prospective markers to spot and effectively pick malignant transformed thyrocytes preoperatively. An accurate and finer technique that can be implicated to thyroid nodules being planned for excision is the detection of these immunomarkers on cytology specimens by immunocytology.^4^,^5^

Galectin-3, plays a pivotal role in processes like cell proliferation, cell-cell interaction, and cell-matrix adhesion mainly via binding to glycoproteins. Especially nuclear Gal-3 is apparently linked with normal cell proliferation.^6^-^8^

Considering the shortcomings of traditional FNAC during diagnosis of thyroid neoplasms and the need of a simple test based on an amalgamation of FNAC and different markers for improving preoperative diagnosis of thyroid nodules, Galectin-3 was employed as a marker in 60 patients who presented with solitary thyroid nodules.

## METHODS

A cross sectional (descriptive and comparative), includes 60 cases of solitary thyroid nodules was conducted at the Departments of Pathology King Edward Medical University/Mayo Hospital and PGMI/General Hospital Lahore. The Purposive convenient sampling technique was used to complete this study in two years i.e. from June 2012 to July 2014.

The ethical committee of the University of Health Sciences, Lahore, approved the study protocol. After taking the consent of the patient, FNAC was done and two smears and a cellblock were prepared. The FNA smears and cellblocks slides were stained with Haematoxylin and Eosin. Formalin fixed cellular aspirate specimen (cell block) was placed in an automated tissue processor (Model RH-12 EP SAKURA Fine Technical Company Limited, Tokyo, Japan) for 17 hours. After processing, tissue embedding was done using paraffin wax. Paraffin embedded cellblocks were kept for Galectin-3immunostaining.

Immunohistochemical staining of the sections prepared from the cellblocks was done.^9^ A labeled antibody is used to identify the antigen of interest in tissue sections. Peroxidase-anti peroxidase immune complex method or the biotin-avidin immunoenzymatic technique is used. Antigen retrieval is carried out to increase the sensitivity of the technique.^10^

The cells that displayed a brownish nuclear/cytoplasmic stain were considered immunopositive.^11^ A positive immunoreactivity was considered when 10% of the tumor cells stained positively for the antibody.^12^ Scoring of the smears for Galectin-3 reactivity was done as follows:^9^

- No staining of tumor cells (0).
- +1 <10% stained cells.
- +2 10-50% stained cells.
- +3 >50% stained cells.

## RESULTS

Out of these 60 patients, the maximum number of cases 44/60 (73%) had a history of duration of disease of 10-20 months (Table-I). It was found that maximum number of malignant cases (follicular carcinoma + papillary carcinoma) 27/37 (72.9%) were also falling in the group of 10-20 months duration of symptoms (Table-I).

**Table-I:** Association of Galectin-3 expression with benign and malignant Thyroid nodules.

	*Histopathological diagnoses*		*Total*	*%age*

	*Malignant*	*Benign*		
Positive	13	23	36	60
Negative	24	0	24	40
Total	37	23	60	100

P-value <0.001 (highly significant).

All the patients presented with solitary thyroid nodule. The mean size of the nodule was 46.02cm^3^ ± 41.66. The minimum and maximum nodule size was two and 168cm three cms respectively. Number of cases having a size of <20cm^3^ (22/60) was same as the number of cases (22/60) having a size in the group of 20-60cm^3^ and was 36% of the total cases.

**Fig.1:**
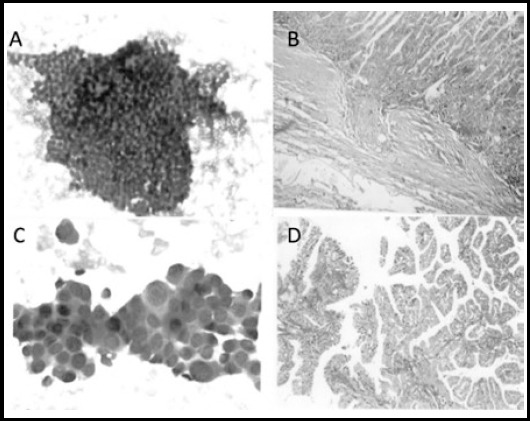
Photomicrograph **A.** FNAC follicular neoplasm Bethesda category IV (H&E stain 200 X), **B.** histological section of follicular carcinoma showing capsular invasion (H&E stain 400 X), **C.** FNAC papillary carcinoma thyroid showing prominent nuclear features (H&E stain 400 X), and **D.** histological section of papillary carcinoma H&E stain 200 X

Most of the malignant case 4/9 (44%) of follicular carcinomas and 12/28 (42%) of papillary carcinomas had a size that fell in the group 20-60cm^3^ while most of the benign cases of follicular adenomas 9/23 (39%) were falling in the group of <20cm^3^ (Fig.1).

The categorization of the smears after cytological diagnosis was done in accordance with The Bethesda System for reporting thyroid cytopathology. 30/60 (50%) aspirates were placed in category IV, 26/60 (43.33%) in categories V and 4/60 (6.67%) patients were placed in category VI. It was found that Category IV was the most common and category VI was the least common category (Fig.1).

Histological diagnosis done on H&E stained sections prepared from the resected nodules showed that among 60 cases of thyroid lesions there were 28/60 (46.6%) cases of papillary carcinoma. Cases of follicular adenoma were 23/60 (38.3%) and 9/60 (15%) cases of follicular carcinoma were reported. However, no case of medullary or anaplastic carcinoma was diagnosed in our study (Fig.1). This was taken as a gold standard.

Galectin-3immunostaining of the sections prepared from the cell blocks was negative in 23/23 benign lesions showing that it was 100% negative in follicular adenomas. Out of 37 malignant cases 24/37 (65%) lesions showed Positivity for this immunomarker while 13/60 (35%) showed negativity for the immunostain. Galectin-3 helped in the preoperative diagnosis of 100% (23/23) follicular adenomas, 75% (21/28) papillary carcinomas and 33.3% (3/9) follicular carcinomas. Overall it helped in reaching a conclusive diagnosis in 78% (47/60) thyroid cytologies (Table-III).

**Table-II:** Expression of Galectin-3 staining in different histologic lesions.

*Galectin-3*	*Histological*			*Total*

	*PC*	*FC*	*FA*	
Positive	7	6	23	36
Negative	21	3	0	24
Total	28	9	23	60

**Table-III:** Predictive value of Galectin-3 in solitary Thyroid nodules.

		*Histopathological diagnosis*		*Total*

		*Malignant*	*Benign*	
Galectin-3 Staining	Positive	True positive(a) 24(40%)	False positive(b) 0(o%)	24
	Negative	False negative(c) 13(21.67%)	True negative(d) 23(38.33%)	36
Total		37(61.6%)	23(38.3%)	60(100%)

***Note:*** Sensitivity =64.87%, Specificity=100%, Positive predictive value=100%, Negative predictive value=63.89%, Accuracy rate=78.33%, Confidence interval = 95% Spearman correlation .644 (*p* –value <0.001)

**Fig.2:**
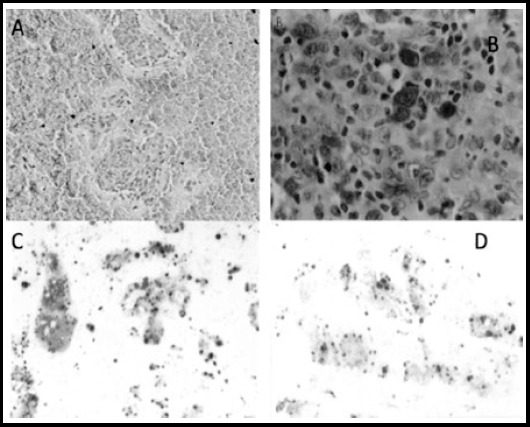
Photomicrograph showing negative control for Galectin-3staining in thyroid follicular cells on FNAC 200X, Photomicrograph showing Positive control for Galectin-3staining in Anaplastic large cell lymphoma 400X, Photomicrograph showing strong positive Galectin-3staining in thyroid follicular cells on FNAC and Photomicrograph showing weak positive Galectin-3staining in thyroid follicular cells on FNAC.

The expression was more common in Papillary carcinoma as compared to follicular carcinoma because out of 21/28 PC (75%) showed positive reaction and only 7/28 (25%) showed a negative reaction while only 3/9 (33%) follicular carcinoma showed positive staining for Galectin-3 antibody and 6/9 (67%) remained negative (Table II-III and Fig.2).

The positivity of Galectin-3 was 65% in malignant cases as compared to benign cases (0%). The difference calculated came out to be highly significant p <0.001 (Table II-III).

The sensitivity and specificity of Galectin-3 in detecting malignant transformed thyrocytes on cytological specimen obtained by FNAC were 64.87% and 100% respectively. The PPV and NPV of Galectin-3 staining were 100% and 63.89% respectively. Moreover, diagnostic accuracy of Galectin-3staining was 78.33%. The confidence interval was calculated to be 95% (Table-III).

## DISCUSSION

Number of cases (benign and malignant) 22/60 having a size <20 cm^3^ was the same as the number of cases with a size of 20-60 cm^3^. Similarly maximum number of papillary carcinomas 12/28 as well as follicular carcinomas 4/9 also fell in the group of 20-60cm.^3^ These results obtained in our research were corresponding to other studies.^13^-^15^ Our findings differ slightly with Basharat et al, who found in their study the size range of thyroid nodules to be 9 cm in greatest dimension.^1^

Nevertheless no noteworthy association between size and duration of growth and the presence of malignancy was found. The findings are consistent with findings of other studies^16^,^17^ who reported that size of nodule and interval of growth of nodule are not helpful for predicting or excluding thyroid malignancy.

Various reporting systems for thyroid cytology have been adopted for the last three decades but none of them was related to the prognosis of disease and patient’s outcome. These reporting schedules were least informative due to variability of sensitivity and least reproducibility. The introduction of the new simplified Bethesda system for reporting Thyroid cytopathology into six categories logically relates to the prognosis of thyroid diseases and increases the reproducibility of diagnosis.^18^ Bethesda Cytopathology Reporting system can help with a better patient’s outcome due to proper clinical management of thyroid swellings and saves patients from unnecessary thyroid surgery. The use of standardized categorical systems for FNAC reporting can make results easier to understand for clinicians and give clear indications for therapeutic action.^19^-^21^

Adopting the above mentioned reporting system the inclusion criteria in our study were, the cytopathology categories IV, V and VI. In our study the most frequent was the category IV with 30/60 (50%) showing follicular neoplasm or showing a risk factor for a follicular neoplasm with specification for Hürthle cell (oncocytic) type. The next commonly seen patients belonged to category V 26/60 (43.3%), showing morphology indicating a risk of malignancy including papillary carcinoma, medullary carcinoma, metastatic carcinoma, or lymphoma. Category VI showing malignant cytology was the least commonly seen class with 4/60 (7%). This class comprised of papillary thyroid carcinoma, medullary thyroid carcinoma, poorly differentiated carcinoma, undifferentiated (anaplastic) carcinoma, carcinoma with mixed features, squamous cell carcinoma, metastatic carcinoma and lymphoma.

On histopathology there were 23/60 (38.3%) cases of follicular adenomas. 28/60 (46.6%) cases were of papillary carcinoma while 9/60 (15%) cases were of follicular carcinoma. None of the tumor revealed the histology of anaplastic or medullary carcinoma.

Amongst these, the most frequent one was papillary carcinoma and it was followed by follicular adenoma. The incidence of higher number of papillary carcinoma than follicular carcinoma is correspondent with other excerpts in the literature.^22^,^23^ This also shows a relationship with the actuality that Papillary carcinomas comprise 80% while follicular carcinomas 10-15% of all thyroid malignancies.^24^

The outcome of a study conducted in, 2008 shows that the immunohistochemical expression of Galectin-3 can serve as a highly specific marker of malignancy. Nevertheless its sensitivity in differentiating malignant from benign thyroid neoplasms is somewhat less.

In our study, Galectin-3 was 100% negative in follicular adenomas because all 23 cases were not reactive on staining with this antibody. Out of 37 malignant cases 24/37 (65%) lesions showed positivity for this immunomarker while 13/60 (35%) showed negativity for the immunostain. With a 65% positivity of Galectin-3 in malignant lesions and 0% positivity in benign cases the difference is highly significant and the *p-* value came out to be < 0.001 which was highly significant.

The negativity does not mean that the cytopathology report should be considered 100% benign because some of the malignant neoplasms were missed by Galectin-3 test and showed a false negative reactivity. Technical issues can be accredited to some of these diagnostic malfunctions at any rate. These can be improved partially by additional specific guidance and committed workshops on the use of Galectin-3 expression testing.

However, a positive expression was always seen in malignant lesions suggesting that positivity of Galectin-3 is a strong indicator for the presence of malignancy in a thyroid aspirate and negates the chance of it being a benign lesion as 0% false positive results were observed in the present study.

The expression was more common in Papillary carcinoma as compared to follicular carcinoma because out of 28 PC, 21(75%) showed positive reaction and only 7 (25%) showed negative reaction while out of 9 follicular carcinoma only 3 (33%) showed positive staining for Galectin-3 antibody while 6 (67%) remained negative. In our research, the positivity of Galectin-3 showed a disseminate cytoplasmic/nuclear staining in most cases of PTC. This included both the classical and follicular variant. The finding is consistent with the study of Aiad et al. according to which Galectin-3 expression was markedly lower in FC as compared to PC.^11^

The follicular thyroid carcinomas also demonstrated the positive expression of Galectin-3 by immunohistochemistry but much less as compared to PTC. Our findings are consistent with Fischer and Asa.^24^

In the present study Galectin-3 helped in the preoperative diagnosis of 100% (23/23) follicular adenomas on FNAC. The result is consistent with a study of Raggio et al., (2010) which also claimed that the immunostain provided a help in the cytological diagnosis of 100% (31/31) follicular adenomas.^25^ As far as papillary thyroid carcinoma is concerned our study aided in the diagnosis of 75% (21/28) smears of PCs. Coming to the pre-operative diagnosis of follicular carcinoma the present study was of a help in 33% (3/9) thyroid aspirates.

According to this study the sensitivity of Galectin-3was found to be 64.87% and specificity of staining was found to be 100%. The negative predictive value was found to be 63.89% while the positive predictive value was 100%. The accuracy rate was recorded as 78.33%. The results were quite similar to the results of a study conducted by Herraiz et al.^13^ where the sensitivity and specificity of Galectin-3 was found to be 60% and 100% respectively.

## CONCLUSION

We recommend Galectin-3 as a supplementary immunostain to be used in in difficult cytologic smears and for differentiation of benign from malignant thyroid lesions especially a hyperplastic papilla from PTC and FA from the follicular variant of PTC. We propose the use of Galectin-3 immunostaining in preoperative FNAC of thyroid nodule more than ever to evaluate the indeterminate cytology and to avoid unnecessary aggressive surgical interference in benign lesions.

### Author’s Contribution

**AM** was the main author who did her MPhil research on this topic.

**MHB** supervised the research.

**IUQ** supervised the thesis.

**AM** is responsible and accountable for the accuracy or integrity of the work.
